# Preoperative Opioid Use Disorder Predicts Prolonged Ventilation, Central Line Placement, and Major Anesthesiology Complications After ACDF Surgery: A Big Data Analysis of 180,000 Cases

**DOI:** 10.3390/jcm14186661

**Published:** 2025-09-22

**Authors:** David Maman, Maneesh Nandakumar, Yaniv Steinfeld, Yaron Berkovich

**Affiliations:** 1 Carmel Medical Center, Haifa 3436212, Israel; yanivsteinfeld@gmail.com (Y.S.); yaron.berkovich@gmail.com (Y.B.); 2 Faculty of Medicine, Technion Israel Institute of Technology, Haifa 2611001, Israel; 3 Logan Hospital, Brisbane, QLD 4131, Australia; maneesh.nan@gmail.com

**Keywords:** anterior cervical discectomy and fusion (ACDF), cost-effectiveness, opioid use disorder (OUD), spine surgery outcomes

## Abstract

**Background:** Opioid use disorder (OUD) has emerged as a growing public health challenge, increasingly affecting surgical populations. While anterior cervical discectomy and fusion (ACDF) is a common spinal procedure with known perioperative risks, the specific impact of preoperative OUD on anesthesia-related complications in ACDF remains poorly studied. **Methods:** We analyzed adult patients undergoing elective single-level ACDF between 2016 and 2022 using the Nationwide Inpatient Sample (NIS) database. Patients with and without OUD were identified using ICD-10 codes. Propensity score matching was applied to adjust for baseline differences. Primary outcomes included prolonged ventilation, central line placement, and major anesthesiology complications. Secondary outcomes included total charges, length of stay, and number of procedures. A cost-effectiveness analysis of universal preoperative urine drug screening was also performed. **Results:** Among 178,215 patients undergoing ACDF, 1.5% had documented OUD. Following propensity matching, OUD patients had a significantly increased risk of prolonged ventilation (>24 h and >96 h), central line placement, blood transfusion, feeding tube insertion, and major anesthesiology complications. OUD patients experienced longer hospital stays (7.9 vs. 2.7 days), more procedures (4.2 vs. 3.0), and higher total charges ($139,207 vs. $82,179; all *p* < 0.01). The estimated excess cost attributable to OUD per surgical patient was $855, compared to a $75 screening cost. **Conclusions:** Preoperative OUD is associated with significantly increased perioperative risk, ICU-level intervention, and healthcare costs in ACDF patients. These findings support systematic preoperative screening and multidisciplinary management for patients with OUD undergoing spine surgery.

## 1. Introduction

Opioid use disorder (OUD) has rapidly become a major public health burden, fueled by a nationwide opioid epidemic [1,2]. According to the 2022 National Survey on Drug Use and Health, approximately 2.1 million Americans aged older than 12 years met criteria for opioid use disorder, reflecting a substantial public health burden, and rates of opioid-related overdose deaths continue to rise [3]. This crisis increasingly impacts surgical patients, as many individuals with chronic opioid use eventually present for operative procedures. Recent data indicate that nearly one in four patients presenting for surgery is taking opioids preoperatively [4]. Moreover, the prevalence of diagnosed OUD among surgical inpatients more than doubled from 0.58% in 2010 to 1.41% in 2018 [5]. These trends illustrate a growing influx of opioid-dependent patients into perioperative care, heightening concerns for anesthetic management and postoperative outcomes. Emerging evidence across surgical specialties suggests that preoperative opioid exposure adversely affects recovery [6]. In orthopedic surgery, for example, preoperative opioid use has been linked to increased postoperative complications in a dose-dependent manner [4].

Spine surgery candidates are often chronically exposed to opioids for pain management before intervention; indeed, patients undergoing anterior cervical discectomy and fusion (ACDF) have higher rates of opioid use than the general population [7]. Consistent with broader guidelines in spine care, which report higher complication rates in patients on long-term opioids [8], it is plausible that OUD specifically confers even greater perioperative risk in this context. However, until now, there has been a paucity of large-scale data examining how a formal diagnosis of OUD impacts ACDF surgical outcomes [9]. ACDF is one of the most commonly performed spinal procedures, indicated for cervical degenerative disc disease, radiculopathy, or myelopathy. While generally safe, ACDF carries perioperative considerations that may be exacerbated by OUD. For instance, patients with OUD or heavy opioid tolerance might require higher opioid doses for adequate anesthesia and pain control, increasing the risk of respiratory depression, difficult weaning from ventilation, and other anesthetic complications. OUD is also frequently accompanied by other comorbidities that could complicate recovery.

Despite these concerns, the relationship between OUD and ACDF outcomes has not been clearly delineated on a national scale. Small single-institution studies and indirect evidence have hinted at worse outcomes with chronic opioid use in spine surgery, but robust epidemiologic analysis specific to OUD in ACDF has been lacking. Given the escalating intersection between OUD and surgical care, there is a critical need to understand the implications of preoperative OUD in spine surgery and to identify high-risk patients before they enter the operating room. Routine preoperative assessment currently screens for common medical risk factors, but systematic screening for substance use disorders is not uniformly implemented. If OUD is found to be an independent risk factor for major perioperative adverse events, this would underscore the importance of incorporating OUD screening and optimization into standard pre-surgical evaluation. In addition, quantifying the impact of OUD on outcomes like prolonged ventilation and intensive care needs can inform healthcare planning and resource allocation, as these events carry significant cost implications.

Study Objective: In this context, we conducted a big-data analysis of approximately 178,215 ACDF cases to evaluate the association between preoperative OUD and perioperative anesthesiology-related outcomes. We specifically examined whether patients with OUD are at higher risk of prolonged postoperative mechanical ventilation, central line placement, and major anesthesiology complications following ACDF. We hypothesized that OUD would be a significant predictor of increased critical care intervention and complication rates. By leveraging a large national dataset—the largest to date for ACDF on this topic—our goal was to fill an important knowledge gap and provide evidence to guide perioperative management. Ultimately, confirming a strong link between OUD and adverse outcomes would highlight the need for systematic preoperative OUD screening and tailored anesthetic strategies (e.g., opioid-sparing techniques and enhanced monitoring) to improve safety, optimize postoperative care (including ICU utilization), and potentially reduce costs.

### Research Questions

Does a history of opioid use disorder worsen clinical outcomes in patients undergoing ACDF?

## 2. Methods

### 2.1. Data Source, Data Access, and Processing

This study utilized data from the Nationwide Inpatient Sample (NIS), the largest inpatient dataset in the world and the most up-to-date national database available. The NIS is maintained by the Healthcare Cost and Utilization Project (HCUP) and captures a 20% stratified sample of all hospital discharges across the United States, representing over 35 million hospitalizations annually. Data from 1 January 2016 to 31 December 2022 were used for this analysis, providing a contemporary, high-powered dataset for evaluating outcomes in ACDF surgery among patients with and without OUD. The NIS is publicly available. Access is granted after completion of the HCUP Data Use Agreement and purchase of the dataset. The NIS is provided in a standardized, de-identified format as fixed-width text (.csv) files accompanied by detailed data dictionaries. Our team downloaded the full 2016–2022 NIS files and loaded them into SPSS (26.0, IBM Corp., Armonk, NY, USA) for processing and statistical analysis. Variables were labeled and formatted according to the HCUP data dictionary. Quality checks were performed, including verification of inclusion/exclusion criteria and frequency review of all study variables. Missing values were handled according to HCUP recommendations: records with missing age, sex, or procedure codes were excluded (<0.1% of cases), while missing comorbidity data were treated as “absent” per HCUP coding guidelines.

### 2.2. Patient Selection and Procedure Code Identification

Patients undergoing elective single-level ACDF were identified using the International Classification of Diseases, Tenth Revision, Procedure Coding System (ICD-10-PCS) codes 0RG10A0 and 0RG10K0. Only adult patients aged 18 years and older were included. To ensure focus on elective procedures, patients admitted emergently or undergoing ACDF as part of trauma care were excluded from the analysis.

### 2.3. Opioid Use Disorder Classification

OUD was defined by the presence of a qualifying diagnosis code from the International Classification of Diseases, Tenth Revision, Clinical Modification (ICD-10-CM) under the F11.x category. Patients were classified as having OUD if any hospital diagnosis field contained an F11.x code during the index admission for ACDF.

### 2.4. Baseline Variables and Comorbidities

Baseline variables collected included patient age, sex, and primary payer type (Medicare, Medicaid, private insurance, self-pay, or other). Comorbidities were identified through ICD-10 codes and included hypertension, dyslipidemia, diabetes mellitus, chronic kidney disease, obstructive sleep apnea, nicotine dependence, obesity, and other medical conditions potentially impacting perioperative risk during ACDF surgery.

### 2.5. Cost-Effectiveness Analysis

A secondary cost-effectiveness analysis was performed to evaluate whether implementing universal preoperative urine drug screening could be justified based on economic impact. The mean excess hospitalization cost attributable to OUD was calculated by subtracting the mean total charge for non-OUD ACDF patients from that of OUD ACDF patients. This difference was multiplied by the observed OUD prevalence of 1.5% among ACDF patients to determine the average excess cost per surgical patient. The excess cost was then compared to the estimated cost of routine urine drug testing, set at $75 per patient, to assess whether screening would result in net healthcare system savings.

### 2.6. Ethical Considerations

This study utilized de-identified, publicly available data and was therefore exempt from institutional review board (IRB) approval. Informed consent was not required, as no protected health information was accessed or analyzed during the course of the study.

## 3. Results

### 3.1. Trends in Opioid Use Disorder Among ACDF Patients

**Figure 1:**
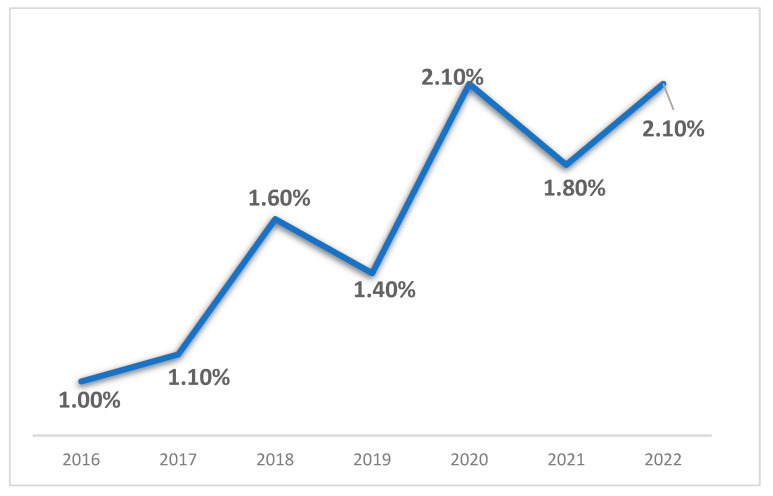
Annual prevalence (%) of opioid use disorder among unmatched ACDF patients, 2016–2022. Error bars show 95% CIs. Trend assessed with Cochran–Armitage (*p* < 0.01).

The prevalence of OUD among patients undergoing ACDF surgery demonstrated a statistically significant upward trend from 2016 to 2022 (Cochran–Armitage trend test, *p* < 0.01) (Figure 1). The proportion of ACDF patients with documented OUD increased from 1.0% in 2016 to 2.1% in both 2020 and 2022, representing a 110% relative increase over the study period. The sharpest year-over-year increase occurred between 2017 and 2018 (1.1% → 1.6%).

### 3.2. Baseline Characteristics of ACDF Patients with and Without OUD

Demographic and clinical characteristics of patients undergoing ACDF (Table 1), stratified by the presence of OUD. Patients with OUD were significantly younger than those without OUD (52.1 vs. 55.9 years, *p* < 0.01), with no significant difference in sex distribution (49.6% vs. 48.2% female, *p* = 0.15). Payer distribution differed markedly between groups (*p* < 0.001), with OUD patients more frequently insured by Medicaid (27.2% vs. 12.4%) and less likely to have private or HMO coverage (26.4% vs. 41.5%).

Several comorbidities differed significantly between groups. OUD patients had higher rates of chronic anemia (6.4% vs. 3.3%, *p* < 0.001), obstructive sleep apnea (11.3% vs. 9.3%, *p* < 0.01), and chronic kidney disease (6.6% vs. 5.2%, *p* < 0.01). The prevalence of dyslipidemia (23.8% vs. 31.7%, *p* < 0.01), nicotine dependence (14.5% vs. 19.5%, *p* < 0.01), and obesity (16.8% vs. 19.0%, *p* < 0.01) was lower in the OUD group. No significant differences were observed in diabetes, thyroid disorders, or Alzheimer’s disease.

### 3.3. Perioperative Resource Utilization Among ACDF Patients with and Without Opioid Use Disorder

We compared perioperative outcomes and healthcare utilization metrics between ACDF patients with and without OUD (Table 2). Patients with OUD had significantly longer hospital stays (7.9 vs. 3.3 days, *p* < 0.01) and higher total hospital charges ($139,207 vs. $96,311, *p* < 0.01). Additionally, they underwent a greater number of procedures per hospitalization (4.2 vs. 3.5, *p* < 0.01).

### 3.4. Propensity Score-Matched Comparison of ACDF Patients with and Without Opioid Use Disorder

To mitigate baseline differences and reduce selection bias, a 1:1 propensity score matching was conducted. This approach allowed for the creation of two well-balanced cohorts of patients undergoing ACDF, one with OUD and one without, matched across key demographic and clinical variables.

Following 1:1 matching (n = 2650 vs. n = 2650), baseline characteristics were well balanced: all SMDs were <0.10 for age, sex, primary payer, and prespecified comorbidities (Table 3), indicating adequate covariate balance for outcome comparisons and ensuring that comparisons in postoperative outcomes would be less confounded by preexisting health differences.

### 3.5. Impact of Opioid Use Disorder on Perioperative Resource Utilization After Propensity Score Matching

We compared perioperative outcomes among propensity score-matched ACDF patients with and without OUD (Table 4). Even after adjusting for baseline differences, patients with OUD experienced significantly greater healthcare utilization. Their mean length of stay was nearly three times longer than that of non-OUD patients (7.9 vs. 2.7 days, *p* < 0.01). Total hospital charges were markedly higher in the OUD group ($139,207 vs. $82,179, *p* < 0.01). The number of procedures performed during the index hospitalization was also significantly greater in the OUD cohort (4.2 vs. 3.0, *p* < 0.01).

### 3.6. Risk of Anesthesia and Intensive Care-Related Interventions After Propensity Score Matching

**Figure 2:**
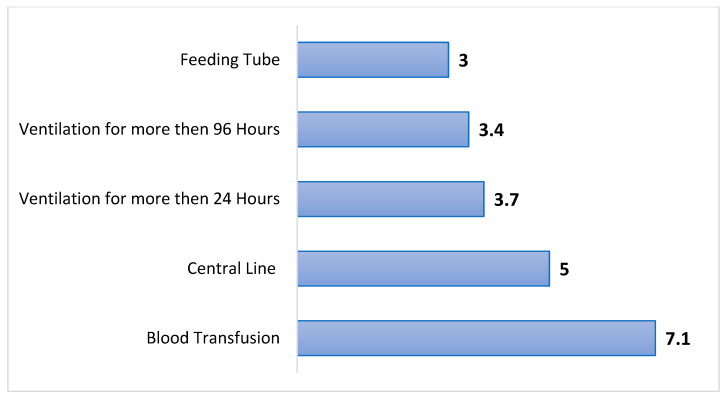
Relative Risk of anesthesia and intensive care-related interventions in propensity score-matched ACDF patients with opioid use disorder.

Following propensity score matching, we assessed the relative risk (RR) of anesthesia and intensive care-related interventions in ACDF patients with and without OUD (Figure 2). OUD patients remained significantly more likely to require multiple critical care interventions. The strongest association was found with blood transfusion (RR = 7.1; 95% CI: 2.8–18.1), followed by central line placement (RR = 5.0; 95% CI: 1.9–13.1). The risk of prolonged mechanical ventilation was also markedly increased in the OUD group, with a RR of 3.7 for ventilation >24 h and 3.4 for ventilation >96 h. Feeding tube placement was significantly more common in OUD patients (RR = 3.0; 95% CI: 1.1–8.3).

### 3.7. Postoperative Complication Risk in Propensity Score-Matched ACDF Patients with Opioid Use Disorder

**Figure 3:**
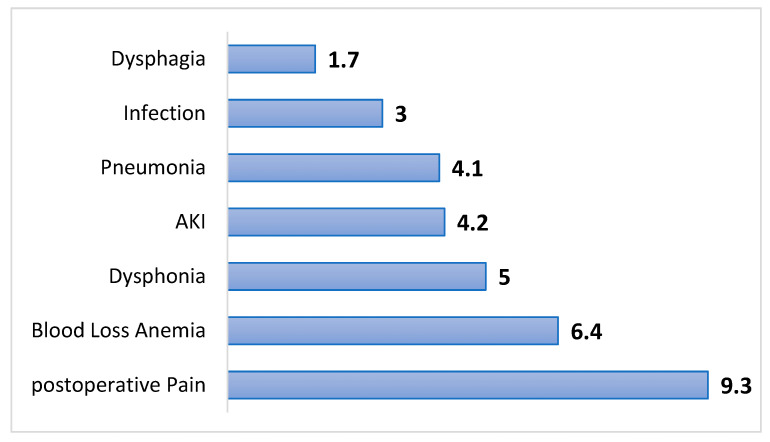
Relative Risk of anesthesia and intensive care-related interventions in propensity score-matched ACDF patients with opioid use disorder.

We evaluated the RR of key anesthesia-related and acute postoperative complications among ACDF patients (Figure 3). OUD patients exhibited a significantly increased risk of several complications, even after adjustment for baseline characteristics. The most elevated risks were observed for urinary tract infection (RR = 9.4; 95% CI: 5.5–16.1) and postoperative pain (RR = 9.3; 95% CI: 6.1–14.1). Other significantly more common complications in the OUD group included blood loss anemia (RR = 6.4; 95% CI: 4.3–9.4), dysphonia (RR = 5.0; 95% CI: 1.9–13.1), acute kidney injury (RR = 4.2; 95% CI: 2.9–6.1), pneumonia (RR = 4.1; 95% CI: 2.3–7.2), and postoperative infection (RR = 3.0; 95% CI: 1.1–8.3). Dysphagia also showed a significantly increased risk (RR = 1.7; 95% CI: 1.4–2.2).

Pulmonary embolism (PE) occurred in 1.5% of patients with OUD, while no PE events were recorded in the matched non-OUD group (*p* < 0.01). Due to the absence of PE cases in the control group, this complication could not be visualized in the figure, as the relative risk is undefined when comparing to zero events.

### 3.8. Economic Justification for Routine Preoperative Opioid Screening in ACDF Patients

**Figure 4:**
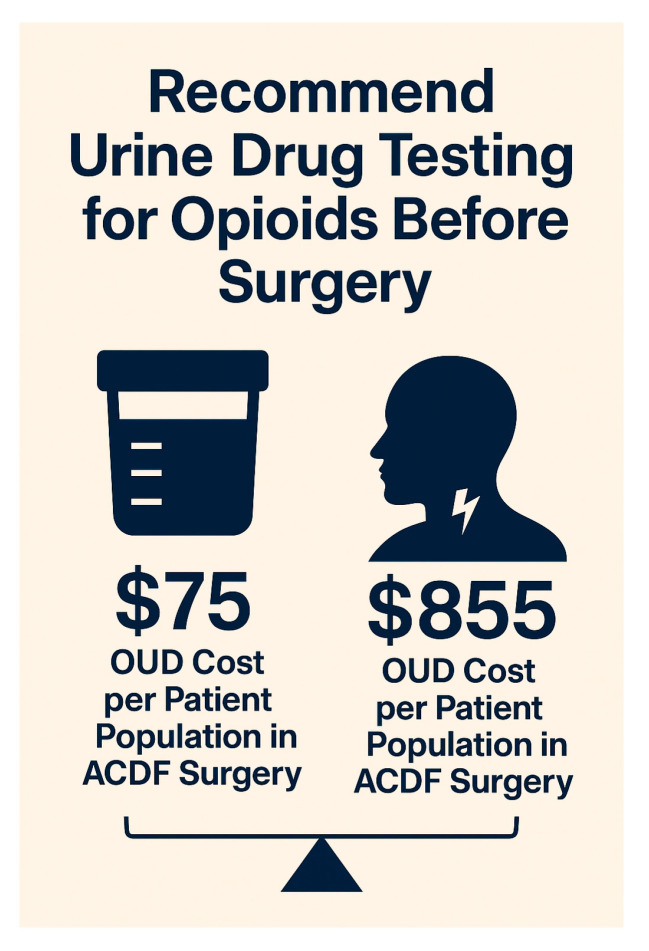
Expected excess hospital charges per ACDF case attributable to OUD (prevalence-weighted) versus a $75 universal screening cost.

In the matched cohort, mean total hospital charges were $139,207 for OUD vs. $82,179 for non-OUD patients (Δ = $57,028). With an OUD prevalence of 1.5%, the average excess charge per ACDF case attributable to OUD is $855 (=$57,028 × 0.015). By comparison, the average cost of a preoperative urine drug screen is approximately $75. As illustrated in Figure 4, this represents more than a 10-fold return on investment per surgical case, even when screening all patients. These findings support the implementation of universal preoperative opioid screening as a cost-effective measure in ACDF surgery.

## 4. Discussion

### 4.1. Key Observations

Our analysis revealed that patients with a history of OUD who underwent ACDF were more likely to experience complications such as prolonged mechanical ventilation, central line insertion, feeding tube placement, and transfusions. These differences persisted even after accounting for other health conditions, indicating a direct association between OUD and increased perioperative risk. The magnitude of effect was clinically significant—for example, length of stay was nearly three times longer and total hospital charges were approximately $57,000 higher in matched OUD patients compared with controls.

### 4.2. How This Compares to Earlier Studies

This study supports earlier research in orthopedic, cardiac, and vascular surgery, where OUD has been linked to poor surgical outcomes [10,11,12]. However, our findings extend this understanding to ACDF, which involves distinct anesthetic and respiratory challenges. This distinction is important, as it emphasizes that OUD poses risks even in surgeries not traditionally associated with high opioid consumption postoperatively. Similar findings have been reported in other surgical populations. Menendez et al. found that opioid abuse or dependence was associated with greater odds of mechanical ventilation (OR 2.3; 95% CI, 2.0–2.5) and longer length of stay (OR 2.5; 95% CI, 2.4–2.5) in orthopedic inpatients [13]. Likewise, Mahamid et al. recently reported that opioid-dependent patients undergoing lumbar fusion had higher rates of respiratory complications (OR 2.17, *p* < 0.001), longer hospital stays (+0.83 days), and USD 17,739 higher mean hospital charges compared with non-dependent patients [14].

### 4.3. Underlying Risk Factors

Patients with OUD often present with several risk factors that complicate surgery. Chronic opioid exposure can lead to respiratory suppression, which delays ventilator weaning [15]. Their need for higher pain medication doses increases the risk of oversedation. Furthermore, immune system suppression associated with opioids may predispose these patients to infections [16]. Co-occurring health and social challenges, like poor nutrition or substance abuse, likely compound these risks.

### 4.4. Practical Considerations for Clinical Teams

The results highlight the need to identify OUD as early as possible in the surgical pathway. A proactive plan might include consultations with pain or addiction specialists, use of medication-assisted therapy, and anesthetic techniques that limit opioid exposure [17]. Postoperatively, these patients may benefit from longer recovery room stays or closer monitoring in an ICU setting, especially if they have a history of respiratory issues [5].

### 4.5. Financial Impact and Health System Implications

The NIS does not contain results of urine drug screens, only ICD-coded diagnoses of opioid use disorder. In clinical practice, screening is typically a qualitative (positive/negative) test, but it serves as an important safety step to identify patients whose OUD might not yet be documented in the medical record or who may not disclose active use. Thus, its value is in detecting previously unrecognized OUD rather than merely confirming known diagnoses. Caring for OUD patients often involves more complex and costly hospital stays. Although introducing screening protocols might seem expensive upfront, they could reduce overall costs by helping prevent avoidable complications [18]. Our findings suggest that something as simple as a routine urine drug screen may be cost-effective when considering the long-term healthcare burden [19].

### 4.6. Strengths and Study Constraints

A key strength of this study is the use of a large, nationally representative database, enhancing the generalizability of our findings across diverse patient populations. Furthermore, we employed rigorous propensity score matching to create well-balanced comparison groups, thereby reducing confounding and improving the validity of the observed associations.

However, several limitations should be acknowledged. First, our analysis relied on administrative coding (ICD-10-CM/PCS), which is subject to misclassification bias and may underreport opioid use disorder or omit important clinical details such as medication regimens and perioperative management strategies. Second, the dataset does not capture important long-term outcomes, including quality of life, functional recovery, or the recurrence of complications after discharge, which limits our ability to assess the full trajectory of postoperative care [20]. Despite robust propensity score matching, residual confounding is possible. The NIS does not capture OUD severity, concurrent benzodiazepine/alcohol use, or social factors that may contribute to perioperative risk. Although the NIS is a rigorously curated national dataset, it is not immune to missingness or coding errors. Our analysis followed HCUP best practices, and missing demographic or procedural data were minimal (<0.1%) and excluded from analysis. Given the large sample size, any residual misclassification is likely to bias toward the null, suggesting our reported associations may be conservative estimates. Future research incorporating quantitative opioid levels or risk stratification by opioid burden could further refine cost-effectiveness analyses.

### 4.7. Areas for Further Study

Looking ahead, more research is needed to explore what happens to these patients after they leave the hospital. Key areas include rates of readmission, chronic pain, and long-term function. It would also be valuable to evaluate specific care models, such as using addiction medicine teams during surgical recovery or adjusting existing recovery protocols to address opioid-related risks. Finally, studies focused on patient experience could help make treatment more personalized and reduce stigma.

## 5. Conclusions

OUD clearly contributes to a higher risk of complications following ACDF. These patients often require more attention and resources throughout their hospital stay. With early identification and better coordination between surgical and addiction care teams, outcomes could be improved. As the opioid crisis continues to affect healthcare, it is essential that surgical planning adapts to recognize and manage OUD more effectively.

## Figures and Tables

**Table 1:** Demographic, insurance, and comorbidity profiles of unmatched ACDF patients, stratified by OUD status.

Variable	No Opioid Use Disorder	Opioid Use Disorder	*p* Value
Patient count (%)	175,565 (98.5%)	2650 (1.5%)	-
Average Age (y)	55.9	52.1	*p* < 0.01 *
Female (%)	48.2	49.6	*p* = 0.15
Medicare (%)	34.4	37	*p* < 0.01 *
Medicaid (%)	12.4	27.2	
Private, including HMO (%)	41.5	26.4	
Self-pay (%)	2.6	5.1	
No charge (%)	0.2	0.6	
Other (%)	8.9	3.8	
Hypertension (%)	43.3	40.9	*p* = 0.17
Dyslipidemia (%)	31.7	23.8	*p* < 0.01 *
OSA (%)	9.3	11.3	*p* < 0.01 *
Nicotine Dependence (%)	19.5	14.5	*p* < 0.01 *
Chronic Anemia (%)	3.3	6.4	*p* < 0.01 *
Osteoporosis (%)	2.3	2.1	*p* < 0.01 *
Parkinson’s Disease (%)	0.6	0.2	*p* = 0.01 *
Alzheimer’s Disease (%)	0.2	0.2	*p* = 0.70
Chronic Kidney Disease (%)	5.2	6.6	*p* < 0.01 *
Congestive Heart Failure (%)	1.1	1.3	*p* = 0.34
Obesity (%)	19	16.8	*p* < 0.01 *
Diabetes Mellitus (%)	20.8	20.2	*p* = 0.41
IBD (%)	0.5	0.6	*p* = 0.91
Thyroid Disorder (%)	11.8	12.3	*p* = 0.45

Abbreviations: ACDF = anterior cervical discectomy and fusion; OUD = opioid use disorder; OSA = obstructive sleep apnea; CKD = chronic kidney disease; IBD = inflammatory bowel disease. *p* < 0.05 was considered statistically significant. Statistically significant differences are marked with an asterisk (*).

**Table 2:** Perioperative resource utilization in unmatched ACDF patients with and without OUD.

	No Opioid Use Disorder	Opioid Use Disorder	Significance
Length of stay mean (Days)	3.3 (Std. deviation 6.2)	7.9 (Std. deviation 12.8)	*p* < 0.01 *
Total charges mean ($)	96,311 (Std. deviation 109,386)	139,207 (Std. deviation 141,288)	*p* < 0.01 *
Number of procedures on this record	3.5 (Std. deviation 2.2)	4.2 (Std. deviation 2.8)	*p* < 0.01 *

Abbreviations: ACDF = anterior cervical discectomy and fusion; OUD = opioid use disorder; LOS = length of stay. *p* < 0.05 was considered statistically significant. Statistically significant differences are marked with an asterisk (*).

**Table 3:** Baseline characteristics of propensity score-matched ACDF patients with and without opioid use disorder.

Variable	No Opioid Use Disorder	Opioid Use Disorder	*p* Value
Patient count (%)	2650	2650	-
Average Age (y)	52.1	52.1	*p* = 0.97
Female (%)	48.3	49.6	*p* = 0.34
Medicare (%)	37	37	*p* = 0.08
Medicaid (%)	26.4	27.2	
Private, including HMO (%)	28.5	26.4	
Self-pay (%)	4.2	5.1	
No charge (%)	0.2	0.6	
Other (%)	3.8	3.8	
Hypertension (%)	40.2	40.9	*p* = 0.58
Dyslipidemia (%)	24.5	23.8	*p* = 0.52
OSA (%)	11.5	11.3	*p* = 0.83
Nicotine Dependence (%)	14.9	14.5	*p* = 0.70
Chronic Anemia (%)	5.5	6.4	*p* = 0.15
Osteoporosis (%)	2.3	2.1	*p* = 0.78
Parkinson’s Disease (%)	0.4	0.2	*p* = 0.20
Alzheimer’s Disease (%)	0.2	0.2	*p* = 1
Chronic Kidney Disease (%)	7	6.6	*p* = 0.59
Congestive Heart Failure (%)	1.1	1.3	*p* = 0.34
Obesity (%)	17.5	16.8	*p* = 0.47
Diabetes Mellitus (%)	20.3	20.2	*p* = 0.86
IBD (%)	0.9	0.6	*p* = 0.11
Thyroid Disorder (%)	12	12.3	*p* = 0.74

Abbreviations: ACDF = anterior cervical discectomy and fusion; OUD = opioid use disorder; PSM = propensity score matching; SMD = standardized mean difference. *p* < 0.05 was considered statistically significant.

**Table 4:** Perioperative resource utilization in propensity score-matched ACDF patients with and without OUD.

	No Opioid Use Disorder	Opioid Use Disorder	Significance
Length of stay mean (Days)	2.7 (Std. deviation 4.6)	7.9 (Std. deviation 12.8)	*p* < 0.01 *
Total charges mean ($)	82,179 (Std. deviation 92,598)	139,207 (Std. deviation 141,288)	*p* < 0.01 *
Number of procedures on this record	3.0 (Std. deviation 2.2)	4.2 (Std. deviation 2.8)	*p* < 0.01 *

Abbreviations: ACDF = anterior cervical discectomy and fusion; OUD = opioid use disorder; LOS = length of stay; PSM = propensity score matching. *p* < 0.05 was considered statistically significant. Statistically significant differences are marked with an asterisk (*)

## Data Availability

The original contributions presented in the study are included in the article further inquiries can be directed to the corresponding author.

## Abbreviations

ACDF	Anterior Cervical Discectomy and Fusion
AKI	Acute Kidney Injury
CI	Confidence Interval
CKD	Chronic Kidney Disease
CMS	Centers for Medicare & Medicaid Services
DM	Diabetes Mellitus
HCUP	Healthcare Cost and Utilization Project
HMO	Health Maintenance Organization
HTN	Hypertension
IBD	Inflammatory Bowel Disease
ICD-10-CM	International Classification of Diseases, Tenth Revision, Clinical Modification
ICD-10-PCS	International Classification of Diseases, Tenth Revision, Procedure Coding System
ICU	Intensive Care Unit
IRB	Institutional Review Board
LOS	Length of Stay
NIS	Nationwide Inpatient Sample
OUD	Opioid Use Disorder
OSA	Obstructive Sleep Apnea
PE	Pulmonary Embolism
PSM	Propensity Score Matching
RR	Relative Risk
Std. deviation	Standard Deviation
UTI	Urinary Tract Infection
